# Value Co-creation in Third-Party Managed Virtual Communities and Brand Equity

**DOI:** 10.3389/fpsyg.2020.00927

**Published:** 2020-05-27

**Authors:** Natalia Rubio, Nieves Villaseñor, MªJesús Yagüe

**Affiliations:** Finance and Marketing Department, Universidad Autónoma de Madrid, Madrid, Spain

**Keywords:** co-creation, virtual community, brand equity, co-innovation, value

## Abstract

Value co-creation continues to be a key issue in the era of marketing 4.0. Despite an increasing amount of research on value co-creation, there is still a lot of ambiguity in the use of certain terms. For example, different names are used to refer to the same concept. Even though the concept of co-innovation or co-production has nearly the same meaning as co-creation, there are certain differences between them that must be clarified. In addition, another difficulty found in the literature is that the co-creation concept is frequently applied to different objects of study, such that it might be dealing with brand value co-creation, co-creation of experience, co-creation attitude, etc. In order to clarify these issues, this paper presents a brief review of the relevant literature on value co-creation.

## Introduction

At present, profound changes are taking place in the markets that are revolutionizing the relationships between consumers and companies, among which the management of big data should be mentioned. Virtual communities are a good example of this, since they owe their success in part to the storage of large amounts of data from individuals, which companies analyze to offer products and services that best meet their needs and thus achieve a competitive advantage. Marketing 3.0 tools based on social networking sites help organizations to increase consumer engagement by analyzing changes in their behavior and promoting dialogue and interaction with them that ultimately bring about a strong bond between the consumer and brands, generating what is called brand equity (Martínez-Cañas et al., 2016). However, according to a recent study based on a series of in-depth interviews with marketing leaders, organizational success will require engaging more deeply with digital transformations to maintain their level of competitiveness. For example, one of these key transformations is represented by virtual reality, which favors the coordination and integration of resources between companies and consumers and improves the perceptions of value-in-use of the latter (Boyd and Koles, 2019). Therefore, in the paradigm of marketing 4.0 and tools 4.0, co-creation remains a fundamental activity for generating value for both organizations and consumers (Gómez-Suárez et al., 2017). One of the fundamental metrics for quantifying the value that organizations obtain is their brand equity or the preference these obtain among consumers. Traditionally, research on co-creation has been attached to the service-dominant logic literature (hereinafter SDL) (Vargo and Lusch (2004, 2008), while brand equity has belonged to brand literature. This study combines both research trends, relating value co-creation and brand equity. Previous studies already indicate that consumers who are involved in co-creation processes have higher levels of loyalty, commitment, and recommendation toward organizations and brands (Casaló et al., 2010; Ariño et al., 2011; Fiol et al., 2012; Agag and El-Masry, 2016).

In the era of marketing 4.0, virtual communities will continue to be an ideal study environment, since they allow the analysis of large amounts of data and favor the co-creation process (Healy and McDonagh, 2013). This paper presents a review of the literature on value co-creation, fundamentally applied to digital environments.

## Conceptual Background: Co-Creation Value

Co-creation value is a fundamental concept in the theory and practice of marketing since the apparition of SDL (Vargo and Lusch, 2016), whose meaning needs to be contrasted in specific digital contexts (Yi and Gong, 2013). Among the most widely accepted definitions of co-creation are as follows: (1) Value co-creation is the joint creation of value by the company and the customers, allowing the customers to co-construct the service experience to suit their context (Prahalad and Ramaswamy, 2004). Subsequently, given that the co-creation of value not only affects the bilateral relationship between the consumer and the company, the definition has been transformed to incorporate the multiple agents involved in the process: and (2) Value co-creation describes the way actors behave, interact, interpret, experience, use, and evaluate propositions based on the social construction of which they are a part (Ranjan and Read, 2016).

The first studies on co-creation assimilated this concept to that of co-production, defined as the participation of the consumer in some of the phases of the development of new products, mainly applied in leading brands (Hoyer et al., 2010; Tynan et al., 2010).

Likewise, the close relationship established between consumers and service providers during the service favors the collaboration between actors (Revilla-Camacho et al., 2015; Cossío-Silva et al., 2016), such that the study of co-creation in the services literature has also been frequent (Chen et al., 2012), mainly in the tourism sector (Mathis et al., 2016). An example of this is the involvement of the users in their experience, when they design, together with the service provider, the activities of a trip, the places to visit, etc. Value co-creation can also occur when users collaborate with organizations in the recovery of a service, since participation for the satisfactory resolution of a problem generates user satisfaction (Roggeveen et al., 2012).

There is currently consensus that co-creation is a multidimensional concept. Its dimensions are grouped around two categories: (1) the ability of the participants to co-create value and (2) their disposition to do so. According to Merz et al. (2018), the first of the categories comes from the literature on engagement and refers to the resources that the participating agents voluntarily offer so that co-creation can take place, such as their knowledge, skills, creativity, and network. This group of dimensions is what Ranjan and Read (2016) understand as co-production, which incorporates variables such as knowledge, equity, and interaction. The second category of dimensions, the favorable disposition toward co-creation, is based on the relationship literature, referring to the motivation of the actors to participate in the co-creation process, and refers to variables such as passion, commitment, and trust. Ranjan and Read (2016) call this second category value-in-use with dimensions of experience, personalization, and relationship. According to Verleye (2015), the dimensions that define the co-creation experience are hedonic experience, cognitive experience, social/personal experience, and pragmatic/economic experience.

Closely linked to value co-creation, but not to be confused with it, are co-creation behaviors. The first authors who measured these behaviors were Yi and Gong (2013), distinguishing between customer participation behavior and customer citizenship behavior. The variables that define the former are information seeking, information sharing, responsible behavior, and personal interaction, and those that determine the latter are feedback, advocacy, helping, and tolerance. According to Rubio et al. (2019), in subsequent studies, these behaviors have been modified depending on the application context (brand communities, digital platforms, and social networks) (Tonteri et al., 2011; Tsai and Pai, 2013; Vernette and Hamdi-Kidar, 2013; Chen et al., 2014; Xu and Li, 2015; Chou et al., 2016; Hu et al., 2016). For example, video watching, video commenting, video producing, and video sharing are co-creation behaviors specific to video-sharing communities (i.e., Hu et al., 2016). Creating groups and/or events, participating in them, sending and answering invitations to friends, and visiting other users’ profiles are behaviors characteristic of social networks (Chen et al., 2014). It should be noted that while there are authors who argue that the search for information is not a co-creation behavior (e.g., Tsai and Pai, 2013), there are other studies that support the opposite thesis (Yi and Gong, 2013; Hu et al., 2016).

Finally, co-innovation is another co-creation behavior stressed by studies of value co-creation in digital environments (Sánchez et al., 2013; Vernette and Hamdi-Kidar, 2013; Bugshan, 2015). Co-innovation as a co-creation behavior is related to users’ participation in contributing ideas, such as for example new product/service modalities, ways to improve them, identification of new users, new moments of consumption, and trends.

Table 1 shows a review of value co-creation definitions and value co-creation scales.

**TABLE 1:** Value co-creation definitions and measurement scalers.

Co-creation definitions	Co-creation dimensions
The process by which stakeholders and organizations jointly create value from products and brands (Prahalad and Ramaswamy, 2004; Merz et al., 2018).	(1) Customer-owned resources: knowledge, persuasion/skills, creativity, and network/connectedness (2) Customer motivation: passion, commitment, and trust
Co-creation is defined as the enactment of interactional creation across interactive system environments (afforded by interactive platforms), entailing agency engagements and structuring organizations (Ramaswamy and Ozcan, 2018)	Not defined
Co-creation is defined as a joint, collaborative, concurrent, peer-like process of producing new value, both materially and symbolically, through the voluntary contributions of multiple actors resulting in reciprocal well-being (Vargo and Lusch, 2016; Busser and Shulga, 2018)	(1) Meaningfulness (2) Collaboration (3) Contribution (4) Recognition (5) Affective response
Co-creation implies that the value exchange is not only defined by the supplier but also negotiated through the exchange of resources between providers, users, and other co-creators (Tommasetti et al., 2017)	(1) Cerebral activities (2) Cooperation (3) Information research and collation (4) Combination of complementary activities (5) Changes in habits (6) Co-production (7) Co-learning (8) Connection
Co-creation process describes the way actors behave, interact, interpret, experience, use, and evaluate propositions based on the social construction of which they are a part. Value can extend into future processes beyond the instant realm of exchange or without the “direct” intervention of another party (e.g., through use, social relation, and joint construction) (Ranjan and Read, 2016)	(1) Co-production: knowledge, equity and interaction (2) Value in use: experience, personalization, and relationship
Co-creation describes the customer as an active participant and collaborative partner in relational exchanges, through involvement in the entire service value chain (Yi and Gong, 2013)	(1) Customer participation: information seeking, information sharing, responsible behavior, and personal interaction (2) Customer citizenship: feedback, advocacy, helping, and tolerance

In short, co-creation behaviors can be classified into different levels (Vernette and Hamdi-Kidar, 2013), starting from a more basic level determined by the search for information, a second level defined through interaction with other actors, creating content and generating feedback, and finally a high level of co-creation, in which participants carry out co-innovation activities. Regardless of the level, the different co-creation behaviors produce positive affective responses, among which should be noted the bond with the brand or the brand equity (Zhang et al., 2015; Busser and Shulga, 2018; González-Mansilla et al., 2019; Omar et al., 2020).

Following Correia Loureiro et al.’s (2019) methodology, 40 papers were reviewed. As a starting point, two seminal articles in the marketing field were analyzed to clarify the theoretical context on value co-creation: Prahalad and Ramaswamy (2004) and Vargo and Lusch (2004). These articles are selected based on their number of citations. This initial process helped us as a starting point when creating search strings for value co-creation (search string: value co-creat^∗^). Two leading databases, Web of Science and Scopus, are employed to get access to the articles. Inside these databases, articles with titles and/or abstracts containing the term value co-creation are searched. The review is restricted to peer-reviewed articles and recent articles (from 2013 to 2019) based on SDL theory.

## Conclusion

Value co-created between actors in the service system is a central premise of SDL (Busser and Shulga, 2018). While value co-creation has received great attention both conceptually and empirically in the marketing literature, it is still necessary to narrow down the meaning of certain concepts related to value co-creation. In this paper, the terms co-production, co-creation, and co-innovation have been delimited, as close to each other, but which present certain differences both in their conceptualization and in their measurement. The difficulty of addressing the meaning of value co-creation is that it is a complex construct, specific to the context and agent centric, and therefore, there are multiple interpretations and various approaches to its measurement (Busser and Shulga, 2018). Likewise, it has been noted that the value co-creation concept has been applied to different objects of study, for example, brand value co-creation (Tajvidi et al., 2017) and co-creation of experiences in the tourism sector (Frias-Jamilena et al., 2017). From a conceptual point of view, the value co-creation process occurs between multiple agents that belong to the same network and that participate directly and indirectly in activities for their mutual benefit and to improve the vitality of the network. As mentioned in the paper, this means that value co-creation can occur between organization and consumer (Yim et al., 2012), between consumers in a virtual community (Chou et al., 2016), between consumers and the virtual community (Rubio et al., 2019), between organization and employees (Dean et al., 2016), and between organizations (Hein et al., 2019). It should also be noted that although value co-creation has been studied as a process of value creation, value can also be destroyed through interactions between the different actors in the network, when they accidentally or intentionally misuse their own resources or those provided by other users, acting in an unexpected or inappropriate way (Harris et al., 2010).

In short, in the era of marketing 4.0, where the importance of big data is remarkable, the virtual communities are a fantastic context for the analysis of large amounts of data provided by their users during the co-creation process, since through these communities, stakeholders share knowledge, skills, competencies, etc. In addition, the virtual community literature suggests that members of communities share consumption experiences and favor attachment to brands, since they passionately show their opinions about them (Merz et al., 2018). This contributes to favoring the brand equity of both the products/services on which comments are made in the virtual community and the brand equity of the virtual community itself, because the more comments there are, and the more reliable and realistic they are and the greater the preference for said community expressed by users.

As limitations, this paper employs two databases of scientific articles. Thus, employing other databases, articles in other languages, and chapters or working papers would enrich our findings. In addition, future research on value co-creation should include other concepts such as open innovation and stakeholder engagement.

## Author Contributions

All authors listed have made a substantial, direct and intellectual contribution to the work, and approved it for publication.

## Conflict of Interest

The authors declare that the research was conducted in the absence of any commercial or financial relationships that could be construed as a potential conflict of interest.
